# Protection conferred by typhoid fever against recurrent typhoid fever in urban Kolkata

**DOI:** 10.1371/journal.pntd.0008530

**Published:** 2020-08-17

**Authors:** Justin Im, Md. Taufiqul Islam, Deok Ryun Kim, Faisal Ahmmed, Yun Chon, K. Zaman, Ashraful Islam Khan, Mohammad Ali, Dipika Sur, Suman Kanungo, Shanta Dutta, Sujit K. Bhattacharya, Gordon Dougan, Kathryn E. Holt, Florian Marks, Jerome H. Kim, Firdausi Qadri, John D. Clemens

**Affiliations:** 1 International Vaccine Institute, Seoul, Republic of Korea; 2 International Centre for Diarrheal Disease Research, Bangladesh, Dhaka, Bangladesh; 3 Johns Hopkins Bloomberg School of Public Health, Baltimore, Maryland, United States of America; 4 National Institute of Cholera and Enteric Diseases, Kolkata, West Bengal, India; 5 Department of Medicine, University of Cambridge, Cambridge, United Kingdom; 6 Department of Infectious Diseases, Central Clinical School, Monash University, Melbourne, Victoria, Australia; 7 London School of Hygiene and Tropical Medicine, London, United Kingdom; 8 Fielding School of Public Health, University of California at Los Angeles, Los Angeles, California, United States of America; George Washington University School of Medicine and Health Sciences, UNITED STATES

## Abstract

We evaluated the protection conferred by a first documented visit for clinical care of typhoid fever against recurrent typhoid fever prompting a visit. This study takes advantage of multi-year follow-up of a population with endemic typhoid participating in a cluster-randomized control trial of Vi capsular polysaccharide typhoid vaccine in Kolkata, India. A population of 70,566 individuals, of whom 37,673 were vaccinated with one dose of either Vi vaccine or a control (Hepatitis A) vaccine, were observed for four years. Surveillance detected 315 first typhoid visits, among whom 4 developed subsequent typhoid, 3 due to reinfection, defined using genomic criteria and corresponding to -124% (95% CI: -599, 28) protection by the initial illness. Point estimates of protection conferred by an initial illness were negative or negligible in both vaccinated and non-vaccinated subjects, though confidence intervals around the point estimates were wide. These data provide little support for a protective immunizing effect of clinically treated typhoid illness, though modest levels of protection cannot be excluded.

## Author summary

Recurrent episodes of typhoid fever can occur in individuals, particularly those living in typhoid endemic regions. Natural typhoid infection should, in theory, offer a degree of protection against future typhoid, however, this protection has only been assessed in a limited number of studies, none of which were conducted recently nor in the context of a carefully controlled clinical trial. The findings from our study, which were based on data from a cluster-randomized trial of Vi polysaccharide vaccine in Kolkata, India, suggest that previous typhoid fever does not provide an appreciable level of protection against future typhoid disease. This finding is important for informing mathematical modelers who predict transmission dynamics within a population and influence the design of effective vaccine intervention strategies.

## Introduction

Typhoid fever is a systemic bacterial infection caused by *Salmonella enterica* serovar Typhi (*S*. Typhi) and is responsible for significant levels of morbidity and mortality in low- and middle-income countries. Global burden of disease studies have estimated that 14.3 million cases of typhoid fever occur annually [1], and case-fatality rates have dropped to <1% since the era of antibiotics ushered in a series of highly effective treatment options [1, 2]. Better understanding of the genetic elements conferring resistance against antibiotics in emergent *S*. Typhi lineages has been made possible by whole-genome sequencing, and the potential for resistant clades to spread locally and globally has been well-documented [3–5]. Consequently, antimicrobial therapy as a means to limit severe disease and death may become critically compromised. Although the typhoid endgame will ultimately be shaped by widespread and lasting access to clean water and improved standards of sanitation, strategic use of existing vaccines, including typhoid conjugate vaccine, must be made a priority as a shorter-term solution [6].

The parenteral Vi capsular polysaccharide vaccine and the oral live-attenuated Ty21a strain are safe and effective but will soon be supplanted by a promising, parenteral Vi-tetanus toxoid conjugate vaccine (Vi-TT), which the WHO prequalified in 2018 and recommended for use in children 6 months to adults 45 years of age in endemic regions [6]. This conjugate vaccine has several potential advantages over earlier vaccines, including extending protection to younger children and inducing long-lasting protection in all age groups [7], and documented protective efficacy of 82% in children between 9 months and 16 years of age after one year of follow-up [8].

The Vi-TT vaccine has the potential to dramatically reduce the burden of typhoid fever in vulnerable populations. However, the optimal strategy for vaccine introduction will be greatly assisted by predictions of dynamic transmission models of typhoid incidence that capture the interplay between natural and vaccine-induced protection. Such predictions will allow policymakers to make rational decisions on such issues as age at vaccination, the need for catch up vaccination, and the frequency of boosting. To date, predictions by these models have been limited by uncertainty about several assumptions, including the protective effect of natural typhoid infection against subsequent typhoid infection in typhoid-endemic settings [9, 10]. Herein, we present an evaluation of the protection conferred by an initial clinically treated typhoid fever illness against subsequent typhoid fever due to reinfection, taking advantage of the multi-year follow-up of a population with endemic typhoid participating in a cluster-randomized control trial of Vi capsular polysaccharide typhoid vaccine.

## Methods

### Ethics statement

All subjects or their guardians provided written informed consent. The protocol was approved by the Institutional Review Boards of the International Vaccine Institute (Republic of Korea), the National Institute of Cholera and Enteric Diseases (India), and the Indian Council of Medical Research.

### Study site

We conducted the evaluation in urban slums of Kolkata, India, where a cluster-randomized trial of Vi polysaccharide vaccine against typhoid was conducted. Two years before the onset of the trial, in January 2003, a population of approximately 60,000 persons was censused, recording individual and household level demographic and socioeconomic data. This information was subsequently updated at regular intervals in a demographic surveillance system that continued through the vaccine trial, culminating in a close-out census at the end of surveillance, two years after vaccination [11]. Typhoid fever surveillance commenced in the study population from the onset of the two-year lead-in period before vaccination and continued for two years following vaccination.

### Vi typhoid vaccine trial

The cluster-randomized trial of Vi capsular polysaccharide typhoid vaccine was initiated in 2004 (ClinicalTrials.gov reference number: NCT00125008). A total of 37,673 individuals, excluding pregnant and lactating women, aged ≥2 years were vaccinated with one dose of either: 1) Vi vaccine (Typherix, GlaxoSmithKline) containing 25μg of Vi polysaccharide; or 2) inactivated hepatitis A (HepA) vaccine (Havrix, GlaxoSmithKline). In the surveillance, participants from the study area who presented at one of five study clinics were examined by a study physician, and for each fever visit, defined as a patient who presented with a history of fever lasting at least 3 days, a blood culture was performed. We defined a typhoid visit as a fever visit in which *S*. Typhi was isolated from a blood culture, with the onset of the illness as the date of fever onset.

### Defining typhoid relapse and reinfections

We defined typhoid *relapse* as a second typhoid visit caused by the infecting strain from the first visit and can be understood as a continuation of the first infection. A typhoid *reinfection* was defined as an independent typhoid visit resulting from a new infection from an external source. Because there is no gold standard for distinguishing relapses from reinfections in all instances of recurrent typhoid visits, we defined typhoid reinfection in several alternative ways.

Our primary approach was based on genomic criteria. *S*. Typhi isolates cultured from the first and all recurrent visits were subjected to whole genome sequencing and single nucleotide polymorphism (SNP) analysis as previously described [12]. Primary analysis considered a recurrent typhoid visit to be a reinfection if there were >5 pairwise SNP differences between isolates from the two visits, a definition that has previously been used to distinguish relapse from reinfection for invasive non-typhoidal salmonellosis [13]. Secondary analyses considered two alternative criteria for defining recurrent typhoid as reinfections. First, we considered the susceptibility pattern across 11 tested antibiotics (chloramphenicol, ampicillin, amoxicillin, trimethoprim-sulfamethoxazole, ciprofloxacin, ceftriaxone, nalidixic acid, tetracycline, ofloxacin, aztreonam, amikacin), where the detection of at least one discordant susceptibility result between isolated pairs indicated infection with a new strain. Second, we considered a recurrent typhoid visit to be a reinfection if a period of >30 days intervened between the date of the first typhoid visit and the date of onset of the next typhoid visit. Each definition was considered irrespective of classification in alternative definitions. It should be noted that each of these definitions gave a conservative measure of the risk of reinfection, since second typhoid attacks not meeting the most stringent definition above, given by genomic criterion, could be caused by reinfection of genetically identical *S*. Typhi strains, given that *S*. Typhi evolution is very slow, less than 1 SNP per year [4, 14].

### Analysis

Our primary analysis, which considered the entire dynamic cohort followed from January 1, 2003 until December 31, 2006, estimated the hazard ratio (HR) for the incidence rate of recurrent typhoid due to reinfection, defined by genetic criteria (*vide supra*), in persons in whom an earlier typhoid visit was observed (Cohort 2), relative to the incidence of a first typhoid visit in persons without an earlier detected visit (Cohort 1). All other analyses were considered secondary. Ratios <1 reflected a protective relationship.

**Fig 1:**
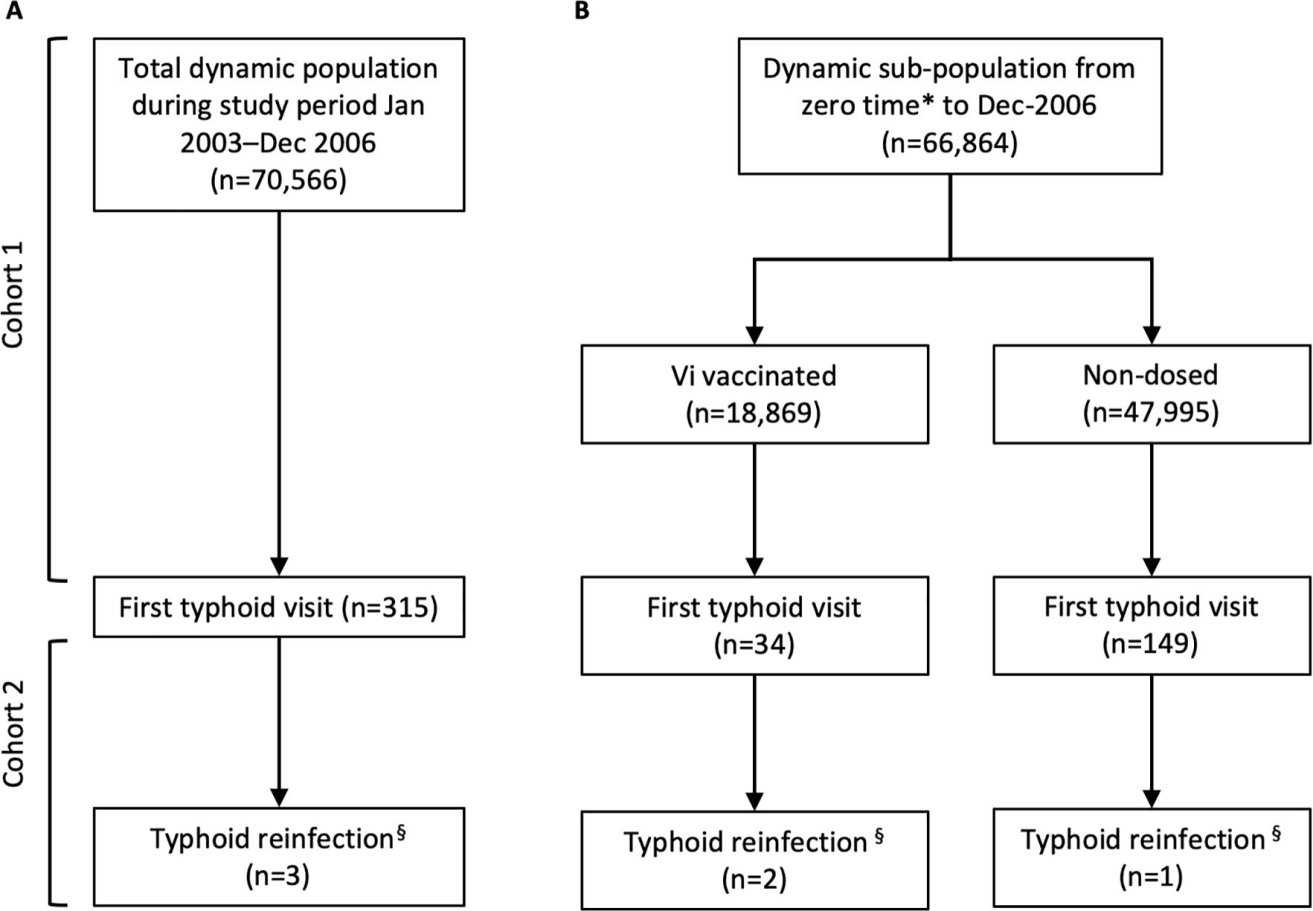
CONSORT diagram showing culture-positive *S*. Typhi first and second visits in A) the total population during the study period of January 1, 2003 until December 31, 2006, and B) sub-populations of Vi-vaccinated and non-dosed individuals from zero time to December 31, 2006. Note: In- and out-migratory populations are not reflected in this diagram. *Given as date of vaccination occurring between November 27, 2004 and December 31, 2004. § Based on pairwise SNP difference >5.

For Cohort 1, we measured the incidence of first typhoid visits for the study population residing in the study area at the study start, January 1, 2003, as well as for persons who moved into the area via in-migration or birth thereafter. Person-days of follow-up for each member of Cohort 1 were calculated from zero time (January 1, 2003, or date of entry into the cohort via birth or in-migration, if after January 1, 2003) until the date of onset of the first typhoid visit date, or one of the right censoring events of out-migration, death or the end of follow-up (December 31, 2006), whichever came first. For Cohort 2, we measured the incidence of recurrent typhoid due to reinfection among those in whom a first visit was recorded, with zero time for follow-up beginning on the visit date for the first visit, and continuing until the date of onset of a reinfection visit, or right censoring event, whichever came first (Fig 1A).

To estimate the HRs relating the rate of recurrent typhoid due to reinfection to the rate of a first typhoid visits in subgroups of Vi vaccinated versus non-vaccinated individuals, we employed a different strategy for defining Cohort 1 and Cohort 2, since vaccination took place between November 27, 2004 and December 31, 2004. Among Vi vaccinees, for Cohort 1, zero time was taken as the date of dosing, with follow-up continuing until the date of onset of the first typhoid visit or the first right censoring event, whichever came first. Cohort 2 measured the incidence of recurrent typhoid due to reinfection among Vi vaccinees in whom a prior typhoid visit was detected, with zero time taken as the date of the first visit and follow-up continuing until the date of onset of a typhoid reinfection visit, or a right censoring event, whichever came first. For the non-Vi vaccinated group, followed during a secular interval comparable to Vi vaccinees, Cohort 1 zero time was the date of vaccination with HepA vaccine, or, among non-dosed individuals, the median date of dosing with either vaccine for other members of the same cluster in the trial if residing in the cluster at the time of vaccination, otherwise birth date or date of migration into the cluster. Zero time for Cohort 2 was the date of the first typhoid visit. Follow-up continued until the date of onset of the first visit (for Cohort 1), the date of onset of the recurrent visit due to reinfection (for Cohort 2), or a right censoring event, whichever came first (Fig 1B).

We fitted generalized Cox models (Andersen-Gill [AG] model), which allow for recurrent events along the timeline for an individual, to estimate HRs, crude and adjusted for potential covariates, and 95% confidence intervals [15]. The AG model is able to evaluate subsequent events which are mediated through time-varying covariates and gives an output indicating the occurrence intensity of a recurrent event. For the primary analysis, the AG HR was adjusted for Vi vaccination status (vaccinated or non-vaccinated) as a time varying covariate and age at zero time, which were independently associated with time to event at p-value <0.10, and protection was expressed as [(1 –adjusted HR) X 100%]. For the subgroup analyses, we fitted models for Vi vaccinees and non-vaccinees separately. We considered p-value <0.05 (two-tailed) as the margin of statistical significance and all modeling was done using the PHREG procedure in SAS/STAT software (SAS Institute Inc., Cary, NC, USA).

## Results

There were 70,566 individuals residing in the study area at any point in time from the study start date to study end (Fig 1A). A total of 315 first typhoid visits were detected in Cohort 1 during the study period, four of which were followed by a second typhoid visit (Fig 1A). Of 315 first typhoid visits, 313 were discharged as outpatients and two were admitted for a duration of 20 and 28 days, respectively. All second typhoid visits were discharged as outpatients. Second typhoid visits occurred in patients ranging from 10 to 33 years of age. Three of four isolate pairs belonged to the H58 haplotype. By SNP criterion, three second typhoid visits were considered to be reinfections with a different strain (Table 1). One second visit fit the reinfection criterion based on antibiotic susceptibility and another based on intervening period definition (Table 1). None of the second visits were classified as reinfection by all defining criteria and one infection pair was not classified as reinfection according to any criteria.

**Table 1:** Classification of recurrent typhoid pairings as due to reinfection based on genomic, antibiotic susceptibility, and intervening period criteria.

ID	NCBI/ENA accession	Haplotype	SNPs^§^ (No. pairwise SNP differences)	Antibiotic susceptibility^∥^ (No. discordant pairs)	Intervening period^¶^ (No. days)
C03551	ERR279346	H58	Reinfection (11)	Relapse (0)	Reinfection (49)
C03891	ERR279347	H58			
C03495	ERR279348	H58	Reinfection (10)	Reinfection (1)	Relapse (19)
C03634	ERR279349	H58			
E02889	ERR279350	H58	Reinfection (9)	Relapse (0)	Relapse (30)
E02990	ERR279351	H58			
E01240	ERR279352	H14	Relapse (5)	Relapse (0)	Relapse (14)
E01303	ERR279353	H14			

§ Pairwise SNP difference >5

∥ Discordance in antibiotic susceptibility between infection pair >0

¶ Intervening period >30 days

The crude incidence of first visits during the entire surveillance period was 0.36 per 100,000 person-days and the crude incidence of all second visits was 0.95 per 100,000 person-days (Table 2). In adjusted analyses, individuals detected with a first typhoid visit were two times more likely to have a subsequent typhoid visit compared to individuals without a prior typhoid visit detected during the study period (Adjusted Protection: -200% (-708, -12)) (Table 2). Using our primary genomic definition of reinfection gave an adjusted protection effect conferred by a first typhoid visit of -124% (-599, 28) (Table 2). Significant protection conferred by first typhoid visit against reinfection, after adjustment for potential confounders, was not detected regardless of which of the three criteria was used to define reinfection, although the antibiotic susceptibility and intervening period criteria gave an estimate that was positive, but low, with wide confidence limits (Adjusted Protection: 26% (-427, 90) and 26% (-429, 90), respectively) (Table 2).

**Table 2:** Protective effect of a first typhoid visit against recurrent typhoid due to reinfection in the total population (n = 70,566).

	No. of individuals	No. of typhoid visits	Person-days †	Incidence per 100,000 person-days	Crude HR ‡	Adjusted Protective Effect (95% CI) ‡
Cohort 1	70566	315	86369890	0.36	Ref	Ref
**All recurrent typhoid visits**						
Cohort 2	315	4	420965	0.95	5.37 (2.00, 14.43)	-200% (-708, -12)
**Recurrent typhoid due to reinfection defined by SNP difference**^§^						
Cohort 2	315	3	421740	0.71	4.00 (1.28, 12.50)	-124% (-599, 28)
**Recurrent typhoid due to reinfection defined by antibiotic susceptibility**^∥^						
Cohort 2	315	1	422584	0.24	1.33 (0.19, 9.46)	26% (-429, 90)
**Recurrent typhoid due to reinfection defined by intervening time**^¶^						
Cohort 2	315	1	422653	0.24	1.33 (0.19, 9.46)	26% (-427, 90)

†In cohort 1, person-days calculated from study start date or date when person entered the study to end date, which is defined as date of study end, out-migration, or onset of the first typhoid visit. In cohort 2, person-days are calculated from the date of first typhoid visit to the date of study end, out-migration, death, or onset of the subsequent typhoid visit.

‡ Fitted a generalized proportional hazard model (AG model). The number of previous infections (0 or 1) was included in the model as a dummy variable; the reference group was the group without a previously detected typhoid visit. The risk was adjusted for Vi vaccination status as a time varying covariate and age at zero time.

§ Pairwise SNP difference >5

∥ Discordance in antibiotic susceptibility between infection pair >0

¶ Intervening period >30 days

Among the four second typhoid visits, two occurred in the Vi vaccine arm and one was in the non-Vi vaccine arm (includes HepA vaccinated and non-dosed) (Fig 1B). One second typhoid visit (E01303) occurred before the period of vaccination and was therefore excluded from vaccination subgroup analyses. Analyses of these subgroups estimated that the crude incidence of first typhoid visit was 0.25 and 0.45 per 100,000 person-days in the Vi-vaccinated group (Table 3) and non-Vi vaccinated group (Table 4), respectively. We identified a very high HR of reinfection (by genomic criterion) in the Vi-vaccinated group with evidence that those with prior typhoid visits were at increased risk for typhoid relative to those without a previously detected typhoid (Adjusted Protection: -3549% (-15473, -755)) (Table 3). In the non-Vi vaccinated group all point estimates for reinfection reflected no protection by an initial typhoid visit (Adjusted Protection: -88% (-1246, 74)), although the wide confidence interval did not exclude a moderate level of protection (Table 4).

**Table 3:** Protective effect of a first typhoid visit against recurrent typhoid due to reinfection in the Vi-vaccinated population (n = 18,869).

	No. of individuals	No. of typhoid visits	Person-days †	Incidence per 100,000 person-days	Crude HR ‡	Adjusted Protective Effect (95% CI) ‡
Cohort 1	18869	34	13705796	0.25	Ref	Ref
**All recurrent typhoid visits**						
Cohort 2	34	2	24624	8.12	74.08 (17.52, 313.21)	-3549% (-15473, -755)
**Recurrent typhoid due to reinfection defined by SNP difference**^§^						
Cohort 2	34	2	24624	8.12	74.08 (17.52, 313.21)	-3549% (-15473, -755)
**Recurrent typhoid due to reinfection defined by antibiotic susceptibility**^∥^						
Cohort 2	34	0	25468	0	--	--
**Recurrent typhoid due to reinfection defined by intervening time**^¶^						
Cohort 2	34	1	24909	4.01	35.56 (4.81, 262.83)	-1640% (-12835, -134)

†In cohort 1, person-days calculated from zero time (date of vaccination) to end date, which is defined as date of study end, out-migration, or onset of the first typhoid visit. In cohort 2, person-days are calculated from the date of first typhoid visit to the date of study end, out-migration, death, or onset of the subsequent typhoid visit.

‡ Fitted a generalized proportional hazard model (AG model). The number of previous infections (0 or 1) was included in the model as a dummy variable; the reference group was the group with no previously detected typhoid visit. The hazard ratio was adjusted for age at date of entry.

§ Pairwise SNP difference >5

∥ Discordance in antibiotic susceptibility between infection pair >0

¶ Intervening period >30 days

**Table 4:** Protective effect of a first typhoid visit against recurrent typhoid due to reinfection in the non-Vi vaccinated population including in-migrants post-vaccination (n = 47,995).

	No. of individuals	No. of typhoid visits	Person-days †	Incidence per 100,000 person-days	Crude HR ‡	Adjusted Protective Efficacy (95% CI) ‡
Cohort 1	47995	149	32868952	0.45	Ref	Ref
**All recurrent typhoid visits**						
Cohort 2	149	1	108912	0.92	4.21 (0.59, 30.13)	-88% (-1246, 74)
**Recurrent typhoid due to reinfection defined by strain**^§^						
Cohort 2	149	1	108912	0.92	4.21 (0.59, 30.13)	-88% (-1246, 74)
**Recurrent typhoid due to reinfection defined by antibiotic susceptibility**^∥^						
Cohort 2	149	1	108912	0.92	4.21 (0.59, 30.13)	-88% (-1246, 74)
**Recurrent typhoid due to reinfection defined by interval** ^¶^						
Cohort 2	147	0	109540	0	--	--

†In cohort 1, person-days calculated from zero time (date of vaccination with placebo or for non-dosed individuals, the median zero time for the cluster of residence at the time of vaccination, or date of entry for in-migrants post-vaccination) to end date, which is defined as date of study end, out-migration, or onset of the first typhoid visit. In cohort 2, person-days are calculated from the date of first typhoid visit to the date of study end, out-migration, death, or onset of the subsequent typhoid visit.

‡ Fitted a generalized proportional hazard model (AG model). The number of previous infections (0 or 1) was included in the model as a dummy variable; the reference group was the group with no previously detected typhoid visit. The hazard ratio was adjusted for age at date of entry.

§ Pairwise SNP difference >5

∥ Discordance in antibiotic susceptibility between infection pair >0

¶ Intervening period >30 days

## Discussion

In this study, we assessed the level of protection associated with typhoid illnesses in patients seeking clinical care against a subsequent visit due to reinfection. Point estimates failed to find a significant level of protection by an initial typhoid illness against typhoid illnesses defined as reinfections using multiple alternative definitions of reinfection. Moreover, our analyses suggested the relationship was different for those who had earlier received Vi polysaccharide vaccine and those who had not, with the former group exhibiting negative protective relationships. The 95% confidence interval upper boundaries were below 0% not only suggesting that there was an absence of protection but that the risk of subsequent typhoid reinfection was actually higher in those with an initial infection. In contrast, non-recipients of Vi polysaccharide, who also had negative values for point estimates of adjusted protection, displayed very wide confidence intervals whose upper boundaries did not exclude moderate protection by an initial visit.

In aggregate, these findings are compatible with previous reports that a natural typhoid infection confers, at best, moderate or incomplete protective effects against subsequent attack [16, 17]. We also observed that the hazard ratios for reinfection were higher in Vi vaccinated compared to non-vaccinated individuals. These data suggest that persons who develop typhoid fever despite earlier Vi vaccination represent a subgroup of non-responders who are especially vulnerable to typhoid, for immune or non-immune reasons. The earlier studies evaluated risk of typhoid in adults from non-endemic settings who were exposed to typhoid during sequential, concentrated outbreaks occurring in a military unit [16] or in the setting of an experimental typhoid volunteer challenge study [17]. These studies did not address the issue of greatest relevance to deployment of new generation vaccines to populations experiencing endemic typhoid: what level of natural protection is conferred by typhoid infection against recurrent infection in a typhoid-endemic population of children and adults followed over a period of several years. This issue which is evaluated in our study, is a key gap in evidence for constructing dynamic transmission models designed to predict the population impact of alternative introduction strategies for new generation typhoid vaccines into populations with endemic typhoid, who account for the vast majority of the world’s typhoid morbidity and mortality.

It is important to discuss the study’s limitations. First, we lacked a gold standard criterion for differentiating typhoid due to reinfection from typhoid due to relapse. However, the consistency of our findings despite use of multiple alternative definitions for reinfection supports the credibility of our findings. In this evaluation, we considered that findings based on genomic criteria, which were more discriminatory, were likely more credible. Secondly, we did not randomly allocate and concurrently follow the two groups under comparison, namely those with previously detected typhoid and those who had not yet been shown to have typhoid. Therefore, those who developed typhoid fever may have been at systematically higher risk of typhoid or were more likely to seek medical care for fever. This may be especially true of those who were vaccine failures, and may not have been completely controlled for in our multivariable models. Counterbalancing this potential bias, however, is the fact that all of our criteria for defining typhoid reinfection were likely underestimates, making our estimates of increased risk conservative, since identical organisms may have caused secondary attacks, given that the low rate of nucleotide substitution in *S*. Typhi would have corresponded to an accumulation of very few SNPs throughout the study [4, 14]. As well, when we controlled for typhoid seasonality to adjust for unequal exposure to typhoid, we found that our results were unaffected. Additionally, this approach of comparing disease incidence in naturally infected versus non-infected individuals in cohort studies has been successfully used to determine protection naturally conferred by infection in other infectious diseases such as cholera [18] and rotavirus [19].

Thirdly, the number of typhoid reinfections, regardless of the defining criteria, were low in number, and the resulting estimates of protection had very wide confidence intervals. However, confidence intervals for most of our estimates exclude even moderate levels of protection. Fourthly, some typhoid visits that were classified as first visits in our surveillance may in fact have been reinfections, as an artifact of the limited time period for our surveillance, though exclusion of these cases would have lowered the incidence of first visits, again making our analyses conservative. Fifthly, our findings reflect the protection conferred by clinical typhoid, excluding asymptomatic or mildly symptomatic typhoid infections, though it seems counterintuitive that these milder typhoid infections could confer greater protection than clinical typhoid severe enough to prompt patients to seek care. Sixthly, all confirmed typhoid cases were treated with antibiotics according to WHO guidelines, considering antibiotic-susceptibility results. Treatment may have reduced the course of infection thereby impacting immunological development and diminishing the measurable protective effect of natural infection. Lastly, these findings reflect the protection conferred by the particular *S*. Typhi strains circulating in the population under observation during the period of study. Importantly, our analysis does not make provisions for undetected typhoid due to limitations of diagnostic sensitivity of blood cultures, although presumably sensitivity is not impacted by the serial order of visits occurring in an individual.

The biological plausibility of our findings is supported by several observations. Supportive evidence includes the intrinsic ability of *S*. Typhi to down-regulate its own virulence genes to facilitate evasion of human immune signaling pathways [20] and to disrupt host transcription regulation effectively disabling components of the host immune response [21]. Additionally, host characteristics, such as genetic determinants [22, 23], prior pathogen exposure [24], and baseline immune cell presence [25] can lead to a muting of immune responses against *S*. Typhi invasion. In contrast, a murine model using *S*. Typhimurium has shown that immunity developed after infection does indeed offer protection from a second attack, though this relationship may be specific to the murine host or to the non-typhoid *Salmonella* pathogen under study [26]. In a human challenge model, elicited immunity provided, at best, only moderate protection against second attack [17].

In view of the dearth of other contemporary studies of natural protection conferred by typhoid disease in human populations with endemic typhoid disease, our analyses should be considered when parameterizing dynamic transmission models of typhoid fever for the purpose of designing programs for and predicting the impact of alternative introduction strategies for Vi conjugate vaccines in endemic settings. Protection conferred by natural typhoid disease against recurrent disease due to typhoid reinfection was not detected in our cohort analyses. However, in view of the limitations of our study (*vide supra*) additional studies of the protective impact of typhoid fever are needed.
